# Editorial: Sex as a biological variable in the neurocircuitry of motivated behavior

**DOI:** 10.3389/fnbeh.2026.1802383

**Published:** 2026-02-19

**Authors:** Paul Leon Brown, Polymnia Georgiou, Amy Stave Kohtz, Antoniette M. Maldonado-Devincci

**Affiliations:** 1 Department of Psychiatry, Maryland Psychiatric Research Center, University of Maryland School of Medicine, Baltimore, MD, United States; 2 Department of Psychology, University of Wisconsin-Milwaukee, Milwaukee, WI, United States; 3 Department of Psychiatry and Human Behavior, University of Mississippi Medical Center, Jackson, MS, United States; 4 Department of Psychology, North Carolina Agricultural and Technical State University, Greensboro, NC, United States

**Keywords:** dopamine, estrogen, motivation, reward, testosterone

Behavioral neuroscience has consistently turned toward motivation as a central driver of behavior and learning. The brain areas traditionally associated with motivation primarily include midbrain dopaminergic nuclei and their projections to the ventral striatum but also include upstream hypothalamic and amygdalar inputs, downstream basal ganglia targets, and several other sensory, limbic, subcortical, and cortical regions. Signaling through these pathways allows organisms to integrate salient sensory input and translate it into actions that fulfill biological needs (Stuber, 2023).

Disruptions in motivational and reward processing are core features of both psychiatric and neurological diseases, including major depressive disorder, schizophrenia, substance use disorder, and Parkinson's disease. For this reason, translational researchers have modeled several aspects of reward processing in animals to better understand the neurocircuitry that regulates motivated behavior under both normal and pathological conditions. The use of these models to develop better treatments and therapeutics for psychiatric and neurological patients who present with impaired motivation is a continuing goal of behavioral neuroscience (Der-Avakian et al., 2016).

In addition, the prevalence, symptomatology, and prognoses of many of the conditions listed above can vary based on the sex of the individual. Diagnoses of unipolar depression are higher in women, but substance use disorder is more prevalent in men. While schizophrenia is equally common among men and women, men are more likely to experience onset during the late juvenile/early adulthood period and exhibit more severe cognitive symptoms than women. In addition, pharmacological treatment for many psychiatric conditions varies by sex due to differences in physiological makeup (i.e., drug metabolism and clearance) or interactions with circulating gonadal hormones. Consequently, it is vitally important to consider sex as a biological variable in the study of motivation as it relates to these disorders (Gobinath et al., 2017).

New epidemiological findings on sex differences have been driven, in part, by the establishment of the NIH Office of Research on Women's Health and the requirement to include female participants in NIH-funded research in the early 1990s. Pre-clinical research has lagged, although a similar policy was announced in 2014 for NIH-funded animal and cellular research (Clayton and Collins, 2014). These policies have led to increased scientific rigor in mental health research and will undoubtedly inform future treatments for psychiatric and neurological disorders. In recognition of this expanded research, we have organized this Research Topic on the subject of *Sex as a biological variable in the neurocircuitry of motivated behavior* and present here a brief synopsis of its articles.

While sex differences in opioid self-administration have been demonstrated in animal models, these differences may be influenced by the type of opioid used and the self-administration model parameters compared. Harris et al. used a behavioral economics approach to specifically compare the demand elasticity of morphine consumption in male and female rats. While they found that female rats have slower initial acquisition of morphine self-administration, which is consistent with previous work, the authors also demonstrated similar elasticity between sexes.

Incubation of craving is a well-established phenomenon in substance abuse research and is thought to reflect relapse vulnerability. Patel and Loweth showed that the incubation of craving for self-administered oxycodone is elevated in male rats and that, while extended oxycodone administration dysregulates the estrous cycle, there is reduced short- and long-term craving in rats in the estrus phase.

Alcohol use disorder is epidemiologically significant and a disorder that has shown sex differences in prevalence and pattern of development. Faccidomo et al. confirmed that escalation of ethanol consumption is greater in female mice than in male mice after an initial self-administration period. Antagonism of AMPA receptors, implicated in alcohol use disorder, was found to reduce response rates equivalently in both sexes. Shobande et al. used an adolescent intermittent ethanol (AIE) vapor model and found modest, sex- and withdrawal-dependent effects on affective behavior, with metabolomic shifts across serum, fecal, and liver samples most pronounced in male individuals shortly after exposure and diminished with longer withdrawal.

Cocaine is another abused substance whose use patterns can be modulated by estrogen in female rodents; however, it is unknown whether estrogens have a similar effect in male rodents. Alvarado-Toress et al. investigated the role of estrogen in cocaine-seeking behavior in male rats using an aromatase inhibitor. They found that a low dose of fadrozole facilitates the extinction of cocaine-conditioned place preference and diminishes the reinstatement following cocaine self-administration.

Hippocampal function is important for memory retrieval and may alter cue-induced substance use. Berry et al. showed that antagonism of dorsal hippocampal beta-adrenergic receptors alters cocaine-conditioned place preference in a sex-dependent manner. While all receptors contributed to conditioned place preference in male rats, in female rats, beta1 receptors drove retrieval, and beta2 receptors drove acquisition and retention.

Similar to reinforcing stimuli, aversive stimuli also influence motivated behavior. Bell et al. demonstrated that the lateral habenula, an aversion-activated brain nucleus, has differing levels of inhibition on dopamine firing in rats, depending on the sex. The transient inhibition commonly seen with habenular activation is of greater duration in male rats and is more likely to be followed by rebound excitation.

Finally, Kniffin and Briand reviewed several regions of the brain and the role that sex differences in glutamatergic transmission may play in reward. These include differences in synaptic structure, strength, and plasticity. These differences are not uniform across the regions discussed, leading to convergent and divergent mechanisms of glutamate transmission by sex.

Animal research, such as in these studies listed above, will continue to inform how sex differences are incorporated into novel therapeutic treatments for pathological conditions. However, there are caveats to consider. To what degree are sex differences more influential than individual variability? Are the sex differences found in animal (or human) research of sufficient size to be of clinical and epidemiological relevance? How well do the animal models translate to the human condition? There is no doubt that expanding scientific knowledge is laudable, but that knowledge is more impactful if it can lead to targeted treatments that help improve the overall health of the population. By increasing the focus on the influence of sex on motivated behavior, it is our hope that these improvements can be achieved.
